# Adipogenic transdifferentiation reprograms EMT-high PDAC cells into a post-mitotic adipocyte-like state and limits metastasis

**DOI:** 10.1038/s41419-026-08613-4

**Published:** 2026-03-20

**Authors:** Yunzhen Qian, Zhixiu Yan, Junjie Wang, Qi An, Jiamei Luo, Musitaba Mutailifu, Aziguli Tulamaiti, Xue-Li Zhang, Zhi-Gang Zhang, Dong-Xue Li

**Affiliations:** 1 State Key Laboratory of Systems Medicine for Cancer, Shanghai Cancer Institute, Ren Ji Hospital, School of Medicine, Shanghai Jiao Tong University, Shanghai, China; 2 The Affiliated Lianyungang Oriental Hospital of Xuzhou Medical University, Xuzhou, China

**Keywords:** Cancer metabolism, Cancer therapy, Metastasis, Differentiation, Transdifferentiation

## Abstract

Pancreatic ductal adenocarcinoma (PDAC) is a notoriously lethal malignancy with high epithelial-mesenchymal transition (EMT) baseline. EMT is associated with enhanced cell plasticity and contributes to tumor adaption and evolution. EMT programs fuel PDAC invasion, metastasis, and treatment resistance, but directly targeting EMT has yielded limited clinical benefits. Transdifferentiation therapy that exploits cell plasticity and redirects malignant cell fate offers an orthogonal approach beyond pathway inhibition. To validate the feasibility of transdifferentiation in epithelial malignancies such as PDAC, we applied an adipogenesis protocol in seven human PDAC cell lines and distinguished AsPC-1 with intensified adipocyte features (intracellular lipid droplets accumulation, elevated adiponectin, CEBPA, PPARG, FABP4 expression). AsPC-1 was converted into adipocyte-like, post-mitotic cells with lipometabolic (enhanced adiponectin secretion and lipolysis) and phenotypic reprogramming (proliferation inhibition, G1 cell cycle arrest, and EMT key transcription factors downregulation). Multi-omics showed global chromatin compaction and transcriptome-wide repression of EMT and metastatic programs in induced AsPC-1 cells, with suppressed MMPs and TGF-β, indicating diminished metastatic potential. Therefore, we further evaluated the possibility of clinical translation by murine orthotopic and hepatic metastasis models, finding adipogenesis induction reduced primary tumor burden and slowed metastatic progression. The adipocyte-like phenotype in vivo was sustained through one-month observation period following induction drug withdrawal. This study establishes a plasticity-oriented “convert-instead-of-kill” strategy for EMT-high PDAC, suggesting a potential for future studies to investigate rational combinations (e.g., transdifferentiation therapy combined with targeted or immunotherapy) to exploit lineage conversion.

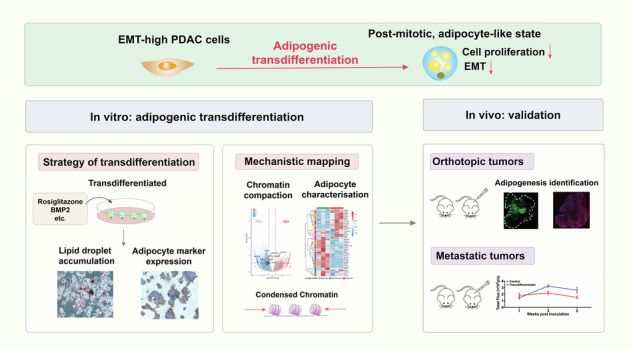

## Introduction

Pancreatic ductal adenocarcinoma (PDAC) is among the deadliest epithelial malignancies, with an annual incidence increase of approximately 1% and a 5-year survival of around 13%, ranking third in cancer mortality, and is projected to become the second leading cause of cancer-related death by 2040 [1]. Despite improvements in radiologic and serologic diagnostics, therapeutic progress has plateaued, and survival remains dismal [2, 3]. High incidence of recurrence and metastasis are major barriers to durable control [4], compounded by chemotherapy resistance [5], a scarcity of actionable targets [6], and a profoundly immunosuppressive tumor microenvironment [7]. These obstacles underscore the urgent need for novel strategies beyond cytotoxicity treatment and single-pathway inhibition.

A defining feature of the pancreatic epithelium, and of PDAC, is phenotypic plasticity. Pancreatic exocrine cells, the progenitors of PDAC, can transdifferentiate into progenitor-like and endocrine-like states [8, 9]. Clinical and pathological observations frequently note intrapancreatic fatty change and adipocyte deposition, implying inherent adipogenic potential within pancreatic derivatives [10–12]. PDAC cells themselves exhibit marked heterogeneity and de-differentiation capacity [13], with a pronounced bias toward epithelial-mesenchymal transition (EMT) driven in part by elevated transforming growth factor beta (TGF-β) signaling and frequent SMAD4 inactivation [14]. Mesenchymal plasticity, especially partial EMT, is increasingly recognized as a prerequisite for dissemination and progression [15, 16]. Molecular taxonomy has identified a quasi-mesenchymal subtype (QM-PDA) characterized by high mesenchymal marker expression and poorer outcomes, encompassing roughly about one-third of PDAC patients [17]. Collectively, these observations suggest that modulating cellular identity rather than merely suppressing EMT-associated pathways [18–20] could be therapeutically advantageous [21].

Cell-fate conversion (transdifferentiation) leverages tumor plasticity by redirecting malignant cells into post-mitotic, lineage-committed states [22]. Differentiation therapy has revolutionized acute promyelocytic leukemia, achieving substantial clinical success with a complete remission rate exceeding 90% [23], and a proof-of-concept study has shown that breast cancer cells with experimentally induced EMT phenotype (via exogenous TGF-β supplement or E-cadherin knockout) can be converted into adipocytes [24]. Given PDAC’s EMT-high biology and apparent adipogenic competence, we hypothesized that enforcing adipogenic transdifferentiation could suppress PDAC growth and dissemination.

In this study, we tested this hypothesis across multiple human pancreatic cell lines and in mouse models. Integrative TCGA/GTEx analysis indicated that PDAC uniquely co-upregulates TGF-β and vimentin, reflecting a high baseline EMT status. Using an adipogenesis-inducing protocol, we identified AsPC-1 as the most susceptible PDAC cell line to adipogenic transdifferentiation, while normal HPDE6.C7 cells also retained adipogenic capacity. Induced AsPC-1 cells acquired adipocyte-like metabolic features, underwent G1 arrest, and displayed EMT reversal. Multi-omics profiling (ATAC-seq and RNA-seq) revealed globally repressed chromatin accessibility and transcriptional quiescence in converted AsPC-1 cells. In orthotopic and hepatic metastasis mouse models, pharmacologic induction of adipogenic transdifferentiation reduced primary tumor burden and restrained metastatic progression.

Together, our findings establish adipogenic transdifferentiation as a mechanistically orthogonal, plasticity-exploiting strategy for PDAC. By converting EMT-biased tumor cells into post-mitotic, adipocyte-like states, this approach may overcome resistance linked to mesenchymal programs and could be particularly relevant for EMT-high subsets such as QM-PDA. Our work provides a rationale and framework for future “convert-instead-of-kill” interventions in PDAC.

## Results

### AsPC-1 is the most permissive PDAC cell line for adipogenic transdifferentiation

Integrated analyses of TCGA and GTEx across multiple human epithelial tissues and their corresponding carcinomas revealed that PDAC uniquely shows concurrent upregulation of both the EMT-inducing factor TGF-β and the mesenchymal marker vimentin (Fig. S1). This signature indicates a heightened EMT propensity in PDAC and provides a rationale for therapies that exploit EMT plasticity.

**Fig. 1:**
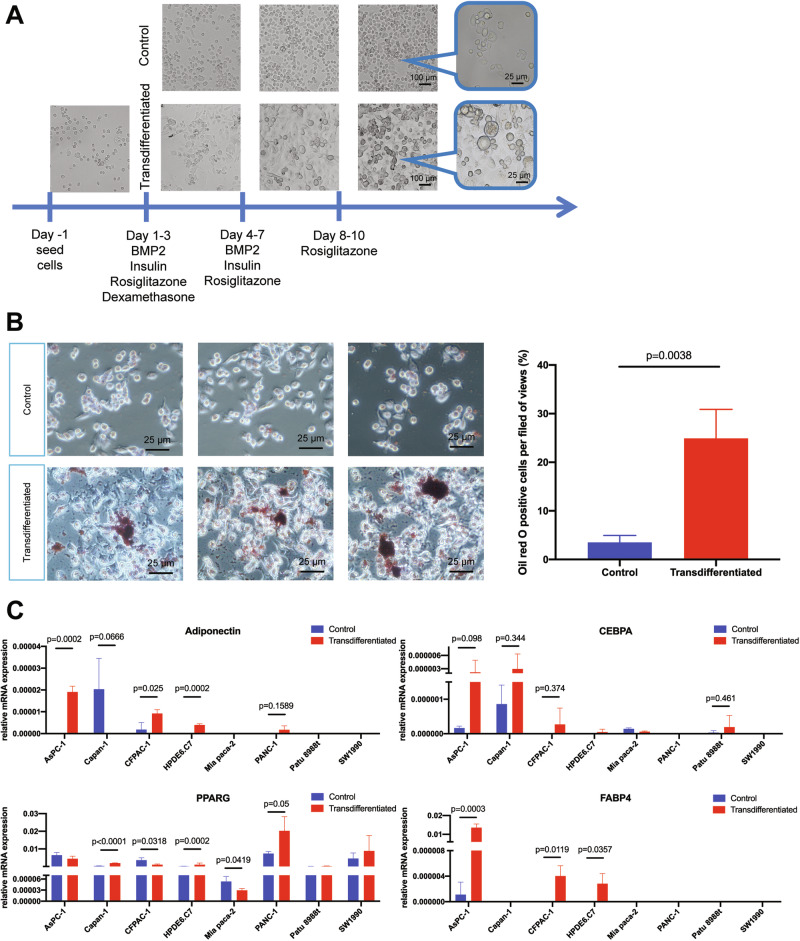
AsPC-1 is adipogenesis-inducible with highest vulnerability among PDAC cell lines. **A** Graphic scheme of adipogenic transdifferentiation induction protocol and microphotographs of control and transdifferentiated AsPC-1 cells at day 0, 3, 7, and 10; Rectangle dialog boxes magnify the field of view from the two groups. **B** Visualization of accumulated lipid droplets by oil red O staining in control and transdifferentiated AsPC-1 cells on day 10 after induction. Five random fields of view were used for quantification, values were presented as the mean ± SD (*n* = 5 per group) and compared by Student’s *t*-test. **C** Relative mRNA expression levels (normalized to *ACTB* expression from the same individual sample) of adipocyte markers *adiponectin, CEBPA, PPARG*, and *FABP4* in eight human pancreatic cell lines. Values were presented as the mean ± SD (*n* = 3 per group) and compared by Student’s *t*-test. Abbreviation: SD Standard deviation.

We applied a 10-day adipogenesis-inducing protocol previously validated in pre-adipocytic fibroblasts and in breast cancer models with experimentally induced EMT [24–26]. In addition to hormonal stimulation by insulin and dexamethasone, a peroxisome proliferator activated receptor gamma (PPARG) agonist, rosiglitazone, was added for transdifferentiation enhancement. Notably, PDAC had a sustained bone morphogenetic protein (BMP) signaling suppression by GREM1 activity to inhibit EMT machinery and maintain epithelial cell fate, while BMP2 treatment can release BMP signaling [27]. BMP2 was thus supplemented in our transdifferentiation protocol to prompt mesenchymal plasticity and promote adipogenesis of PDAC. To account for PDAC heterogeneity, we screened seven human PDAC cell lines (AsPC-1, Capan-1, CFPAC-1, Mia PaCa-2, PANC-1, PaTu 8988t, SW-1990) alongside a normal pancreatic cell line (HPDE6.C7). Phase-contrast microscopy documented characteristic adipocyte-like changes after induction—greater cellular size, increased optical transparency, and a rounder morphology (Fig. 1A). Marker analyses showed line-to-line variability. AsPC-1, CFPAC-1, and HPDE6.C7 exhibited more pronounced conversion, with elevated oil red O positive staining ratio (Figs. 1B and S2) and coordinated upregulation of mature adipocyte markers adiponectin, CCAAT enhancer binding protein alpha (CEBPA), and fatty acid binding protein 4 (FABP4) (Fig. 1C), whereas the other cell lines displayed minimal adipogenic response induction.

**Fig. 2:**
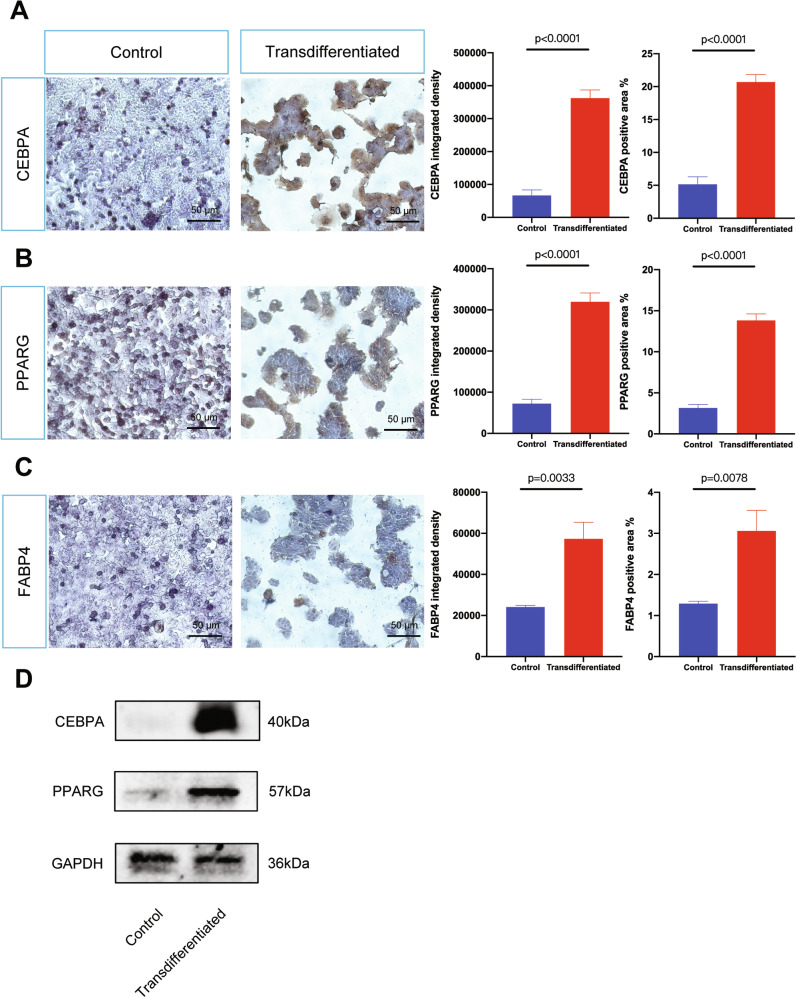
Transdifferentiated AsPC-1 cells express high levels of adipocyte-specific proteins. Visualization and quantification of immunostaining of CEBPA (**A**), PPARG (**B**), and FABP4 (**C**) in control and transdifferentiated AsPC-1 cells on day 10. The protein expression levels were quantified by integrated intensity and positive area of five random fields of view. Values were presented as the mean ± SD (*n* = 5 per group) and compared by Student’s *t*-test. **D** The protein expression levels of CEBPA and PPARG as adipogenesis markers and GAPDH as reference marker in AsPC-1. Abbreviation: SD standard deviation.

Successful induction in HPDE6.C indicates that normal pancreatic cells retain intrinsic adipogenesis competence. Given its superior efficiency, AsPC-1 was selected as the primary model for subsequent studies. Immunocytochemistry (ICC) confirmed robust protein-level increases of CEBPA, PPARG, and FABP4 in induced AsPC-1 cells. Quantification showed significant elevations by integrated density (Fig. 2A–C, CEBPA: 5.44 fold, *p* < 0.0001; PPARG: 4.43 fold, *p* < 0.0001; FABP4: 2.38 fold, *p* = 0.0033) and positive area density (CEBPA: 4.03 fold, *p* < 0.0001; PPARG: 4.38 fold, *p* < 0.0001; FABP4: 2.38 fold, *p* = 0.0078). Immunoblotting corroborated the upregulation of CEBPA and PPARG (Fig. 2D). PPARG expression showed a dissociation between mRNA and protein abundance; a discrepancy may be mediated by post-translational and post-transcriptional regulation.

### Adipogenic transdifferentiation suppresses AsPC-1 proliferation and EMT

**Fig. 3:**
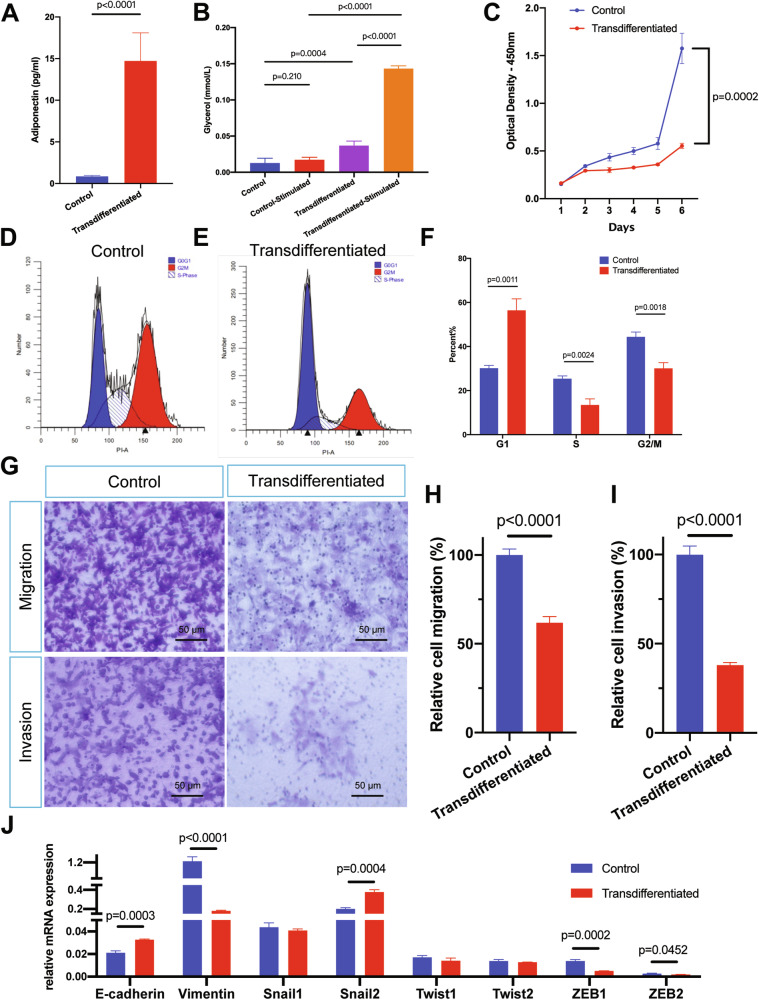
Metabolic reprogramming and phenotypic alteration of AsPC-1 induced by adipogenesis transdifferentiation. **A** Adiponectin secretion levels in control and transdifferentiated AsPC-1 cells after 10 days’ induction. Values were presented as the mean ± SD (*n* = 6 per group) and compared by Student’s *t*-test. **B** Glycerol release from control and transdifferentiated AsPC-1 cells after 10 days’ induction. Isoproterenol was used to stimulate cellular lipolysis. Values were presented as the mean ± SD (*n* = 5 per group) and compared by Student’s *t*-test. **C** Cell proliferation of control and transdifferentiated AsPC-1 was monitored for 6 days during adipogenesis induction. Values were presented as the mean ± SD (*n* = 5 per group) and compared by Student’s *t*-test. **D**–**F** Cell cycle analysis of control **D** and transdifferentiated **E** AsPC-1 after 10 days’ induction. Percentages of cell numbers of G1, S, and G2 phases were plotted as the mean ± SD (*n* = 3 per group) and compared by student’s *t*-test. **G**–**I** Representative fields of Transwell migration and invasion assays for control and transdifferentiated AsPC-1 cells after 10 days’ induction. The Y axis represented relative migration or invasion cell percentage, normalized to the average of control group (set as 100%). Quantification was performed by analyzing five random fields (**H**, **I**), and values were presented as the mean ± SD (*n* = 5 per group) and compared by Student’s *t*-test. The numerous tiny dots are the pores of the Transwell membrane. **J** Relative mRNA levels of EMT markers (*E-cadherin* and *vimentin*) and EMT-TFs (*Snail1, Snail2, Twist1, Twist2, ZEB1*, and *ZEB2*) in control and transdifferentiated AsPC-1 cells. Values were presented as the mean ± SD (*n* = 3 per group) and compared by Student’s *t*-test. Abbreviation: SD standard deviation, EMT epithelial-mesenchymal transition, TF Transcription factor.

Adipogenically transdifferentiated AsPC-1 cells secreted markedly more adiponectin—a adipocyte specific adipokine (Fig. 3A, 17.05 fold, *p* < 0.0001), and displayed robust lipolytic responses to β-adrenergic stimulation (Fig. 3B, 8.25 fold, *p* < 0.0001), indicating acquisition of mature adipocytes-like metabolic function. We next asked whether induction also enforced a post-mitotic state.

Cell growth assays showed pronounced proliferation arrest after induction (Figs. 3C and S3A). Cell cycle profiling demonstrated G0/G1 accumulation (Figs. 3D–F 56.42 ± 5.22% versus 30.19 ± 1.20% in control group). Flow cytometry revealed increased forward scatter (Fig. S3B, C), consistent with the larger cell size seen by phase contrast microscopy. Together, these data indicate terminal like differentiation with curtailed cell-cycle progression. Transwell assays indicated that adipogenesis induction significantly reduced the migration and invasion capacities of AsPC-1 cells (Fig. 3G–I, relative cell migration rate 61.85 ± 3.49%, relative cell invasion rate 37.98 ± 1.48% comparing to control group).

To evaluate EMT status, we quantified canonical markers [28]. Induced AsPC-1 cells upregulated E-cadherin (CDH1) and downregulated vimentin (Fig. 3J). EMT-inducing transcriptional factors (EMT-TFs), zinc finger E-box binding homobox1 and 2 (ZEB1 and ZEB2), were also reduced, indicating a reversal of mesenchymal traits.

It is well established that cross-regulation exists between CEBPA and PPARG [29], and CEBPA is unable to promote adipogenesis in the absence of PPARG [30]. Since our adipogenesis induction cocktail comprised rosiglitazone, a PPARG agonist, we further asked whether rosiglitazone was the key component and PPARG was the central signaling molecule in the adipogenesis process of AsPC-1 cells. We established a “rosiglitazone-omitted” subgroup that underwent the same induction protocol as the transdifferentiated group but without rosiglitazone. The “rosiglitazone-omitted” subgroup displayed partial adipogenic features but was not competent with that of the transdifferentiated group. Consistent with this phenotypic shift difference, Transwell assays revealed the migration and invasion capacities of the “rosiglitazone-omitted” subgroup were intermediate between the control and transdifferentiated groups (Fig. S4).

### Adipogenesis enforces a globally repressed chromatin state and an adipocyte-like transcriptome

**Fig. 4:**
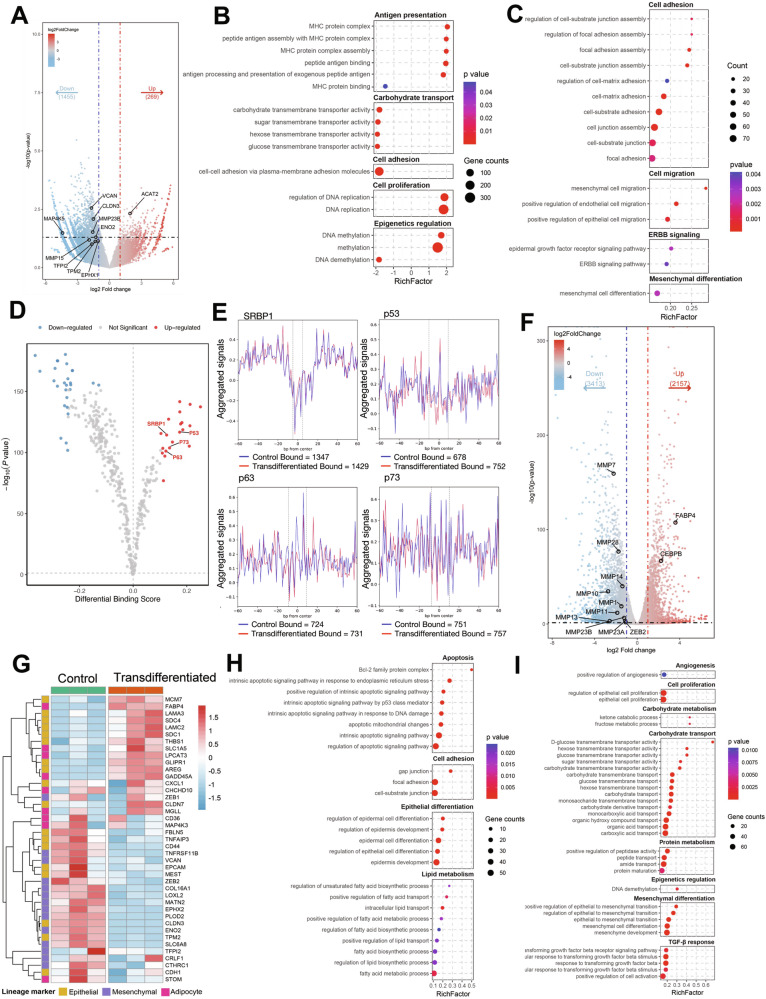
Multi-omics of adipogenesis-induced AsPC-1 cells showed global chromatin compaction and transcriptomic remodeling. **A** Volcano map of differentially accessible regions (DAR) between control and transdifferentiated group (*n* = 3 per group). Left, closed genes in transdifferentiated group (gene counts = 1455); middle, gene regions with insignificant change; right, opened genes in transdifferentiated group (gene counts = 269). **B** GSEA of DARs displayed differentially enriched pathways in the transdifferentiated group. **C** GO term analysis of DARs based on gene counts provided significantly downregulated pathways in the transdifferentiated group, indicating decreased accessibility and reduced transcriptional activity of these gene regions. **D** TOBIAS based differential footprinting analysis. Red dots represented transcription factors (TFs) with elevated binding scores in the transdifferentiated group, and blue dots represented TFs with diminished binding scores. The detailed results were provided in Tables S2 and S3. **E** Aggregated footprint plots of SRBP1 (1347 versus 1429, TF binding sites in control group versus transdifferentiated group), p53 (678 versus 752), p63 (724 versus 731), and p73 (751 versus 757). The dashed lines represented the edges of binding motifs. **F** Volcano map of differentially expressed genes (DEGs) between control and transdifferentiated group (*n* = 3 per group). Left, downregulated genes in transdifferentiated group (gene counts=3413); middle, genes with insignificant change; right, upregulated genes in transdifferentiated group (gene counts=2157). **G** Heatmap of unsupervised hierarchical clustering of 41 differentially expressed epithelial-specific (yellow), mesenchymal-specific (purple), and adipocyte-specific (pink) genes between control (left) and transdifferentiated (right) groups. The original data was normalized using the z-score to indexes. GO term analysis of DEGs based on gene counts showed significantly upregulated (**H**) and downregulated pathways (**I**) in transdifferentiated group, indicating reduced cell proliferation and metastasis. Abbreviation: DAR differentially accessible regions, GSEA gene set enrichment analysis, DEG differentially expressed genes, GO gene ontology, TF transcription factor.

To define the epigenomic and transcriptional basis of these changes, we performed assay for transposase accessible chromatin with high throughput sequencing (ATAC-seq) and transcriptomic sequencing (RNA-seq) in control and induced AsPC-1 cells. ATAC-seq revealed a genome-wide decrease in chromatin accessibility after induction (Fig. 4A). We identified 269 regions with increased accessibility and 1455 regions with reduced accessibility, including multiple matrix metalloproteinases (MMPs, EMT markers). This redistribution is consistent with phenotypic repression of EMT and metastatic programs upon adipogenic conversion. GSEA of differentially accessible regions (DAR) showed enrichment for cell adhesion, antigen presentation, DNA methylation, and DNA replication (Fig. 4B). GO analysis revealed that regions with decreased chromatin accessibility were enriched for terms related to cell adhesion, migration, and mesenchymal differentiation (Fig. 4C), suggesting selective chromatin compaction at these loci. The changes in TF binding activity were then analyzed by TOBIAS based differential footprinting analysis (Fig. 4D). Notably, sterol regulatory element binding protein 1 (SRBP1), p53, p63 and p73 showed significantly elevated TOBIAS differential binding scores, suggesting their reinforced roles in the transcriptional regulation of downstream target genes in the transdifferentiated group and potential participation in driving adipogenesis and tumor repression (Fig. 4E).

Genes with opened chromatin accessibility displayed regulatory transcription through interactions with either activating or repressing TFs, whereas genes exhibiting reduced chromatin accessibility had diminished transcription [31, 32]. Consistent with this, RNA-seq showed a global reduction in gene expression—a hallmark of post mitotic states (Fig. 4F). MMPs were broadly downregulated, suggesting diminished invasive potential. Adipogenesis markers FABP4 and CEBPB showed significant upregulation. Unsupervised clustering of lineage markers demonstrated a clear phenotypic shift: untreated cells grouped with mesenchymal phenotype, whereas induced cells clustered with mature adipocyte signatures (Fig. 4G). GO term analysis of RNA-seq indicated upregulation of apoptosis, cell adhesion, and lipid metabolism pathways (Fig. 4H) and downregulation of cell proliferation, TGF-β response, and mesenchymal differentiation programs (Fig. 4I). Collectively, these multi-omics data indicate that AsPC-1 cells transition from an EMT-high status to transcriptionally quiescent, adipocyte-like cells with reduced cell proliferative and metastatic capacities.

### Pharmacologic adipogenic transdifferentiation restrains PDAC growth and metastasis in vivo

**Fig. 5:**
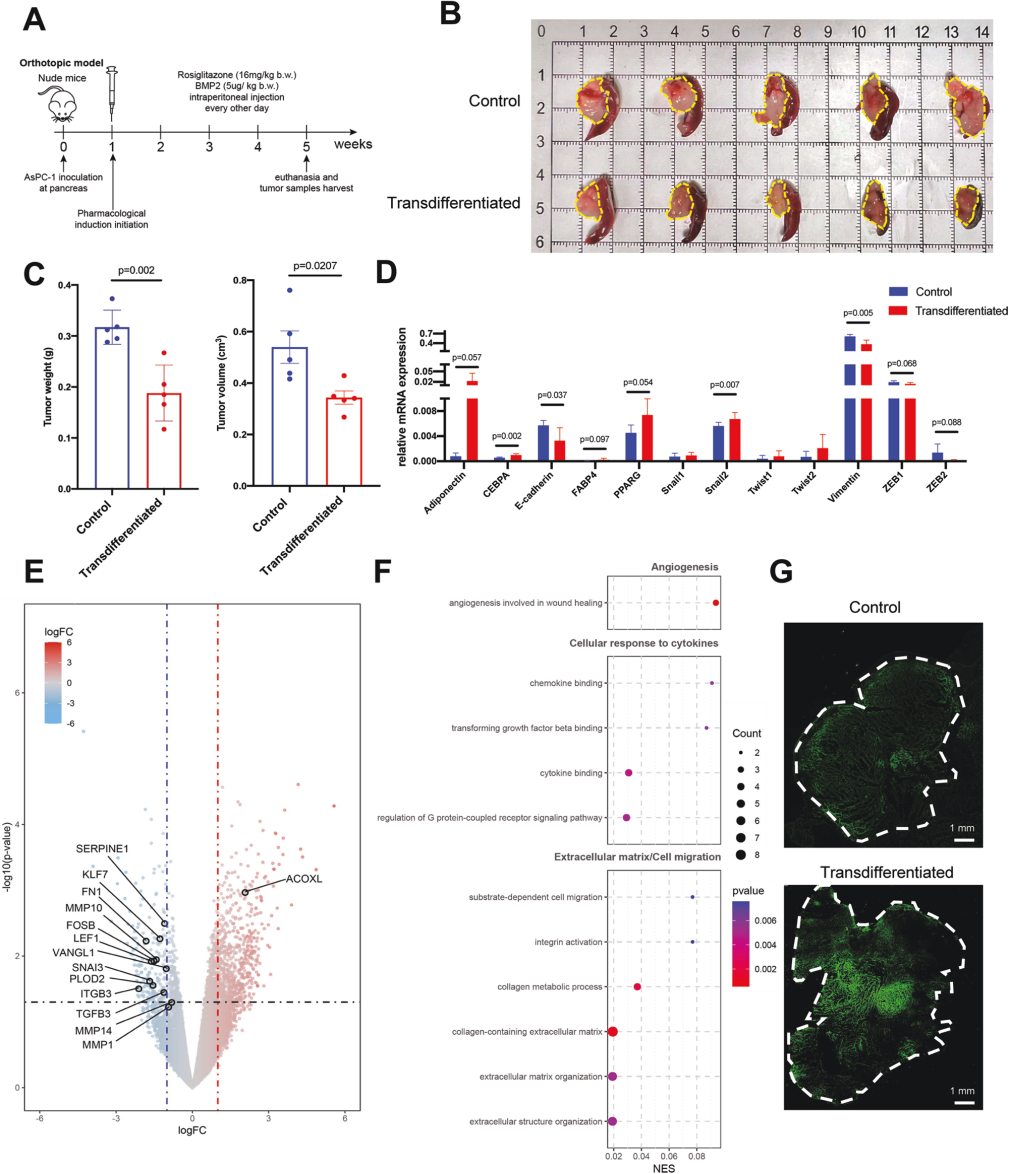
Adipogenic transdifferentiation treatment suppressed orthotopic PDAC tumor growth. **A** Schematic illustration of orthotopic PDAC models workflow. **B**, **C** Orthotopic PDAC samples collected from control and transdifferentiated groups. Tumors were outlined with yellow dashes. Tumor weight and tumor volume were presented as mean ± SD (*n* = 5 per group) and compared by Student’s *t*-test. **D** Relative mRNA expression levels of adipogenesis markers (CEBPA, PPARG, FABP4, and adiponectin), EMT markers (E-cadherin and vimentin), and EMT-TFs (Snail1, Snail2, Twist1, Twist2, ZEB1, and ZEB2) in control and transdifferentiated groups. Values were presented as the mean ± SD (*n* = 5 per group) and compared by Student’s *t*-test. **E** Volcano map of differentially expressed genes (DEGs) between control and transdifferentiated group (*n* = 5 per group). **F** GO term analysis of DEGs based on gene counts showed significantly downregulated pathways in transdifferentiated group, indicating reduced angiogenesis, cellular response to cytokines, extracellular matrix organization, and cell migration. **G** BODIPY detection of lipid droplets in control and transdifferentiated groups from orthotopic PDAC samples. Tumors were outlined with white dashes; a representative view of fields was magnified and displayed in Fig. S6. Abbreviation: PDAC Pancreatic ductal adenocarcinoma, SD Standard deviation, EMT epithelial-mesenchymal transition, TF transcription factor; DEG differentially expressed genes, GO Gene ontology.

Prompted by the in vitro conversion, we tested the therapeutic efficacy in vivo. In orthoptic PDAC models, transdifferentiated group showed significantly reduced tumor weight (0.188 ± 0.055 g versus 0.317 ± 0.033 g, *p* = 0.002) and tumor volume (0.344 ± 0.057 cm^3^ versus 0.540 ± 0.141 cm^3^) compared with controls after four weeks treatment (Fig. 5A–C). Quantitative PCR detected increased expression of adipocyte specific genes together with decreased vimentin and EMT-TFs transcripts (Fig. 5D). We further sequenced the mice orthotopic tumor samples to comprehensively elucidate the transcriptomic alterations induced by adipogenic transdifferentiation in vivo. The transdifferentiated group displayed significantly decreased expression of MMPs, along with an enhanced adipocyte gene signature (Fig. 5E). GO term analysis indicated downregulation of cellular response to cytokines and cell migration (Fig. 5F).

BODIPY staining detected enhanced florescence intensity of intratumoral lipid droplets, and immunofluorescence assays confirmed the elevated expression of adiponectin, CEBPA, PPARG, and FABP4 in transdifferentiation-treated tumors (Figs. 5G and S5, S6). Thus, pharmaceutically forced adipogenesis suppressed orthoptic PDAC progression in vivo.

**Fig. 6:**
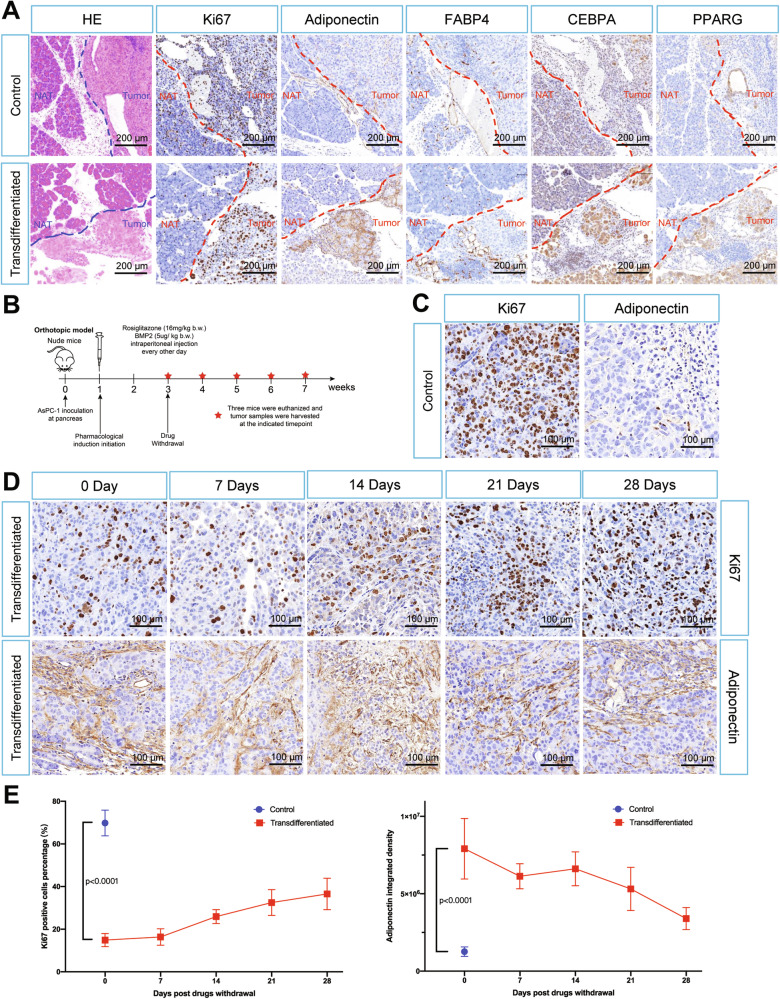
Adipogenic transdifferentiation treatment had specific and persistent influence on orthotopic PDAC tumor. **A** Histopathological analysis of serial sections containing both orthotopic tumors and normal adjacent pancreatic tissues (NAT) from control and transdifferentiated groups (analyzed at 3 weeks post tumor cells inoculation). **B** Schematic illustration of drug withdrawal assays workflow. **C** Representative magnified views of Ki67 and adiponectin immunohistochemistry staining in control group (3 weeks post tumor cells inoculation). **D** Representative magnified views of Ki67 and adiponectin immunohistochemistry staining in transdifferentiated group. Images were arranged in chronological order (left to right), corresponding to sequential time points after induction drug withdrawal (0–4 weeks). **E** Longitudinal analysis of Ki67 and adiponectin immunohistochemistry staining intensity post induction drugs withdrawal (0–4 weeks). Five random fields of view were used for quantification, values were presented as the mean ± SD (*n* = 3 per time point) and compared by Student’s *t*-test. Abbreviation: NAT normal adjacent tissue.

We then examined the specificity of this regimen and durability of the adipocyte-like phenotype in vivo. HE staining and immunostaining indicated no significant morphological changes or enhancement of adipocyte marker gene expression in normal adjacent pancreatic tissues, suggesting the in vivo treatment regimen had limited off-target effects on normal pancreatic tissues (Fig. 6A). By longitudinal comparison of Ki67 staining and adiponectin staining, we proposed the drug induced adipocyte conversion of PDAC cells can maintain its adipocyte-like phenotype for at least four weeks in vivo (Fig. 6B–E).

**Fig. 7:**
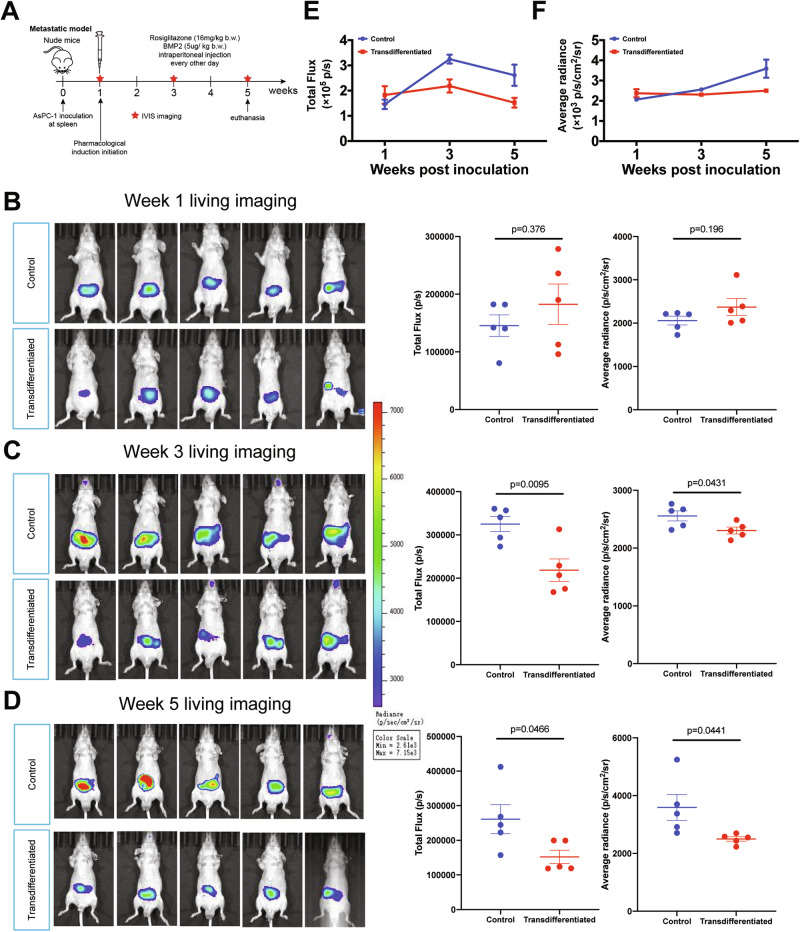
Adipogenesis treatment repressed PDAC metastasis in hepatic metastasis mouse models. **A** Schematic illustration of metastatic PDAC models workflow. Living imaging analysis of PDAC metastatic tumor burden in control and transdifferentiated groups (*n* = 5 per group) at 1-week post-inoculation (**B**) 3-weeks post-inoculation (**C**) and 5-weeks post-inoculation (**D**). The two groups had comparable total flux intensity and average radiance at 1-week timepoint (treatment initiation time-point), and differed significantly at 3-weeks timepoint and 5-weeks timepoint. Values were presented as the mean ± SD and compared by student’s *t*-test. Longitudinal comparison of metastatic tumor burden at 1-, 3-, and 5-weeks post-inoculation based on total flux intensity (**E**) and average radiance (**F**). Values were presented as the mean ± SD (*n* = 5 per group). Abbreviation: PDAC Pancreatic ductal adenocarcinoma, EMT Epithelial-mesenchymal transition, SD Standard deviation. Abbreviation: PDAC Pancreatic ductal adenocarcinoma, SD Standard deviation.

Given the EMT attenuation observed in vitro, we next evaluated metastatic control in PDAC hepatic metastasis models using in vivo living imaging to track tumor burden (total flux intensity and average radiance). Groups were comparable at week 1 post tumor cells inoculation (Fig. 7B, total flux: 1.826 ± 0.782 × 10^6^ versus 1.455 ± 0.417 × 10^6^ photons/second, *p* = 0.376, average radiance: 2.372 ± 0.442 × 10^3^ versus 2.06 ± 0.227 × 10^3^ photon/second/cm^2^/steradian, *p* = 0.198). By week3, significant divergence emerged, the control group displayed an expansion in tumor area (Fig. 7C, total flux: 2.185 ± 0.582 × 10^6^ versus 3.25 ± 0.392 × 10^6^ photons/second, *p* = 0.0095, average radiance: 2.304 ± 0.133 × 10^3^ versus 2.557 ± 0.194 × 10^3^ photon/second/cm^2^/steradian, *p* = 0.0431), and the control group displayed the most regional intensified signal by week 5 (Fig. 7D–F, total flux: 1.522 ± 0.433 × 10^6^ versus 2.611 ± 0.941 × 10^6^ photons/second, *p* = 0.0466, average radiance: 2.501 ± 0.177 × 10^3^ versus 3.589 ± 1.004 × 10^3^ photon/second/cm^2^/steradian, *p* = 0.0441). These results establish pharmacologic induction of adipogenic transdifferentiation as an anti-tumor strategy that suppresses PDAC growth and metastasis in vivo.

## Discussion

The high lethality of PDAC is driven in large part by its aggressive capacity for local invasion and distant metastasis [33]. EMT, a dynamic cellular program, recognizes adhesion and cytoskeletal architecture to promote migration and invasion capabilities [34–36], and also fuels genomic instability that accelerates tumor evolution [15]. Beyond canonical TGF-β signaling, recent studies have identified additional regulators of EMT in PDAC, including PBRM1 loss [37], ATM deletion [38], NSD2 stabilization and KDM2 inactivation [39, 40], underscoring the multiplicity and resilience of EMT control nodes.

Therapeutic efforts to counter EMT have typically pursued three avenues: blocking upstream inducer, disrupting core EMT-TFs (Twist, Snail, ZEB), and eliminating cancer cells with mesenchymal phenotype. Here we leverage a different principle: exploiting tumor plasticity to redirect cell fate. By enforcing adipogenic transdifferentiation, we convert EMT-biased PDAC cells into post-mitotic adipocyte-like cells with reduced proliferative and invasive potential. This approach complements pathway inhibition by acting orthogonally to malignant programs rather than suppressing EMT alone; it respecifies lineage identity. Besides, transdifferentiation protocol comprises differentiation inducer, thereby circumventing additional therapy-associated cytotoxicity. It is plausible that this strategy may be generalized to other EMT-biased tumors, such as kidney cancers [41, 42], as suggested by its upregulation of TGF-β and vimentin (Fig. S1).

Across tested models, three cell lines were responsive to the adipogenic transdifferentiation protocol (AsPC-1, CFPAC-1, HPDE6.C7). The ability of HPDE6.C7 to undergo adipogenic conversion indicates an intrinsic adipogenic competence in normal pancreatic epithelium, consistent with clinical observations of intrapancreatic fatty replacement across pancreatitis, diabetes, and pancreatic cancer [43]. Among PDAC cell lines, AsPC-1 exhibited the highest susceptibility. Its origin from malignant ascites of a metastatic case [44] is congruent with a heightened EMT bias, which we posit facilitates efficient lineage redirection. Notably, lipid droplets in transdifferentiated cancer cell lines appeared as focal clusters, contrasting with the more diffuse distribution pattern seen in transdifferentiated HPDE6.C7 cells (Fig. S2). This difference may reflect dysregulated lipid metabolism dynamics and heterogenous adipogenic response within the cancer cell population.

Mechanistically, our multi-omics data showed global chromatin compaction and transcriptome-wide downregulation after induction, accompanied by repression of EMT-TFs (ZEB1, ZEB2) and mesenchymal programs, aligning with a post-mitotic, adipocyte-like state. TOBIAS footprinting analysis displayed enhanced binding of SRBP1, p53, p63, and p73, indicating these as transcriptional drivers contributing to the lineage conversion and phenotypic alteration in transdifferentiated AsPC-1 cells. Murine models further confirmed that adipogenesis intervention reduced orthotopic tumor burden and curtailed hepatic metastatic progression, indicating that lineage conversion can yield therapeutic benefit in complex microenvironments.

BMP signaling represents a potentially relevant axis for fate control in PDAC. Prior work showed that persistent BMP suppression helps maintain epithelial identity and that GREM1 inhibition of BMP can restore epithelial traits in mesenchymal PDAC cells [27]. While our study was not designed to dissect BMP causality, the responsiveness we observed is compatible with a model in which BMP activity can facilitate adipogenic commitment under permissive conditions. Deconvoluting the role of BMP2/GREM1 in PDAC fate choice—using ligand/receptor perturbation and temporal single-cell profiling—will be an important direction.

Lineage conversion may also recondition tumor-immune interactions. Our GSEA analysis of ATAC-seq DARs indicated enrichment of antigen presentation pathways in induced AsPC-1 cells, suggesting increased chromatin accessibility at loci relevant to major histocompatibility complex I (MHC-I) processing. Aberrantly downregulated cellular surface MHC-I expression is a well-established mechanism of tumor immune evasion. Researchers have developed various methods to restore MHC-I levels, thereby strengthening immune recognition and targeting of tumor cells, such as pharmacological induction [45] and oncolytic virus engineering [46]. Our finding implies that adipogenic transdifferentiation might synergize with immunotherapy by enhancing tumor visibility. This hypothesis warrants functional validation (surface MHC-I quantification, antigen-specific T cell assays, and combination studies with checkpoint blockade).

Combination strategies that co-target oncogenic signaling and plasticity are particularly appealing in PDAC (KRAS mutant rate> 90%). Prior work showed that the MEK inhibitor trametinib can augment adipogenic transdifferentiation [24]. Given trametinib’s extensive evaluation in KRAS-mutant malignancies [47–49], rational combination with adipogenic transdifferentiation merits study—ideally in genomically annotated, EMT-high models—to test whether MEK pathway dampening both sensitizes to fate conversion and stabilized the adipocyte-like state.

This study has limitations. First, responsiveness varied across lines, highlighting inter-tumoral heterogeneity and the need for biomarkers of susceptibility (e.g., EMT-high baseline, TGF-β/vimentin co-upregulation, ZEB1/2 activity, chromatin openness at adipogenic loci). Second, oil red O staining in cellular assays revealed that the transdifferentiation efficiency of AsPC1 was approximately 25%. Although the adipogenesis protocol succeeded in prior researches, its future application in pancreatic cancers may require protocol optimization to enhance efficiency and specificity. Third, adipocytes can be metabolically active participants in tumor ecosystems; future work should exclude adverse paracrine or lipid-fueling effects on non-converted neighbors by spatial transcriptomics and metabolite tracing. Finally, translation will require defining a clinically acceptable induction regimen, delivery, and safety profile (including off-target adipogenesis).

In summary, we provide functional evidence that adipogenic transdifferentiation constitutes a feasible and effective strategy against PDAC, corroborated by in vitro cell lines and in vivo murine models. By enforcing a transcriptionally quiescent, adipocyte-like state and suppressing EMT programs, lineage conversion offers mechanistically orthogonal therapeutic avenue with potential to overcome resistance associated with mesenchymal plasticity. The observed remodeling of antigen-presentation pathways and the precedent for MEK-inhibitor augmentation together outline testable combination therapies, and systematic investigation is warranted to find a biomarker predicting patients responsive to transdifferentiation therapy.

## Subjects and methods

### Online databases

Open access transcriptomics data were sourced from The Cancer Genome Atlas (TCGA) and The Genotype-Tissue Expression project (GTEx) databases using Xena browser (https://xenabrowser.net). Comparison of vimentin and TGF-β expression in TCGA and GTEx databases was performed using GEPIA2 (http://gepia2.cancer-pku.cn) [50], which normalized data by the maximum median expression value across all blocks and then made comparison analysis between normal pancreatic tissues and PDAC.

### Cell culture

The human pancreatic cancer cell lines AsPC-1 (RRID: CVCL_0152), CFPAC-1 (RRID: CVCL_1119), Mia PaCa-2 (Cat#SCSP-568), PANC-1 (Cat#SCSP-535), SW-1990 (Cat#SCSP-5279) were obtained from the National Collection of Authenticated Cell Cultures, Capan-1 (Cat#HTB-79) were obtained from the American Type Culture Collection, PaTu 8988t was obtained from Procell (Cat#CL-0579). The human pancreatic ductal epithelial cell line HPDE6.C7 was obtained from Kyushu University. Cell lines were authenticated by STR and tested for mycoplasma contamination.

AsPC-1 was cultured in RPMI 1640, Capan-1 and CFPAC-1 were cultured in IMDM, HPDE6.C7, Mia Paca-2, PANC-1, and Patu 8988t were cultured in DMEM, and SW1990 was cultured in L15 medium. All cell culture media were supplemented with 10% heat-inactivated fetal bovine serum and 100 U/mL penicillin–streptomycin, and DMEM used for culturing MIA PaCa-2 was additionally supplemented with 2.5% horse serum. All cells were cultured at 37 °C with 5% CO_2_.

In order to visualize and monitor the metastatic load in vivo, AsPC-1 cells were stably transduced with a luciferase-encoding lentivirus prior to their use in the murine PDAC hepatic metastatic models.

### Murine models

Animal experiments have been approved by the Experiment Animal Center, Shanghai Jiao Tong University (Ethical approval number: A2025194). Male BALB/c nu mice aged 6–8 weeks were housed under specific pathogen free conditions, and assigned to experimental groups using simple randomization. All postoperative interventions were provided by staff blinded to the group assignment. Sample size was not predetermined with statistical methods, but based on expected effect size and variability within the sample, as well as cost and feasibility of the experiments.

Orthotopic PDAC models were established by injecting 1 × 10^6^ AsPC-1 cells suspended in 25 μl PBS into the tails of pancreas using a syringe. PDAC hepatic metastatic models were established via intrasplenic injection of 1 × 10^6^ luciferase reporter AsPC-1 cells suspended in 25 μl PBS. To restrict metastatic seeding specifically to the liver, splenectomy was performed after cell administration. In vivo adipogenesis induction was performed one week after tumor cell inoculation. At the designated time points (1-, 3-, 5-weeks post inoculation), potassium D-luciferin (SB-D1009, ShareBio, China) was administered intraperitoneally, and signal was captured using IVIS Spectrum (PerkinElmer, USA) and processed using Living Image 4.7.4 (Binning 8, smooth 11 × 11). Five weeks after inoculation, mice were euthanized, and tumor tissue samples were collected.

To track the long-term fate of adipogenic cells in vivo, adipogenesis induction was maintained for two weeks. Following the withdrawal of the inducing drugs, three mice were euthanized weekly, and their tumor samples were harvested for histopathological analysis.

### Pharmacologically forced adipogenesis

Cells were seeded in 12-well plates (1 × 10^5^ cells per well) and allowed to adhere overnight. Following attachment, the growth medium was refreshed with the adipogenesis induction medium, containing 5 μg/ml insulin [51], 2 μM rosiglitazone (dissolved in DMSO), 1 μM dexamethasone (dissolved in DMSO), and 200 ng/ml BMP2. After three days incubation, dexamethasone was withdrawn; after seven days incubation, insulin and BMP2 were withdrawn. The induction process lasted for a total of 10 days, with the medium being changed every other day. Control cells were treated with medium containing equivalent DMSO. The “rosiglitazone-omitted” subgroup underwent an paralleled induction procedure, differing only in the absence of rosiglitazone.

In murine models, to mitigate potential hormone related adverse effects mediated by insulin and dexamethasone, a streamlined transdifferentiation protocol that omitted both hormonal components was applied [24]. The transdifferentiated group was administered with rosiglitazone (16 mg/kg) and BMP2 (5 μg/kg), while the control group received an equivalent volume of PBS. Mice received intraperitoneal injections every other day until the designated endpoint (drugs withdrawal or euthanasia).

### Lipid droplets staining

In vitro cell samples were fixed and stained by Oil Red O staining kit (R23104, OriLeaf, China) according to the manufacturer’s instructions. Lipid droplets were visualized after color differentiation with 60% isopropanol, and nuclei were counterstained by hematoxylin staining solution (C0107, Beyotime, China). Stained lipid droplets were examined under Axio Observer (ZEISS, Germany).

Murine orthotopic PDAC tissues were rapidly frozen in liquid nitrogen and cryosectioned into 4-μm slides. Sections were then stained with BODIPY (D3922, ThermoFisher, USA) to visualize lipid droplets and subsequently imaged using DMI8 Thunder (Leica, Germany).

### Immunocytochemistry, immunofluorescence, and immunoblotting

Cell samples were fixed with 4% paraformaldehyde; 3% H_2_O_2_ was used to block endogenous peroxidases for 15 min. followed by 2.5% goat serum block for an hour. The following primary antibodies were used: polyclonal antibody against CEBPA (12968-1-AP, Proteintech, USA), polyclonal antibody against FABP4 (12801-1-AP, Proteintech), polyclonal antibody against PPARG (16643-1-AP, Proteintech), and recombinant antibody against Ki-67 (GB1514499, Servicebio, China). The isotype control was ab37415 (polyclonal rabbit IgG, Abcam). Subsequently, a secondary HRP-conjugated antibody (M21002S, Abmart, China) was applied. Coloration was performed using a SignalStain DAB Substrate Kit (8059, Cell Signaling Technology, USA).

Frozen sections of tumor tissues were blocked and incubated with primary antibodies following the same procedure as mentioned above. Subsequently, the slides were incubated with a secondary antibody (Ab6564, Abcam) for one hour, mounted using Antifade Mounting Medium with DAPI (P0131, Beyotime), and visualized using DMI8 Thunder.

Cell samples were lysed using RIPA (P0013B, Beyotime) and separated by 15% SDS-PAGE. Subsequently, proteins were transferred onto 0.45 μm nitrocellulose filter membranes and blocked with 5% milk. Membranes were then incubated with different primary antibodies as aforementioned and GAPDH monoclonal antibody as endogenous control (MA5-15738, ThermoFisher). Secondary antibody was applied after. Protein bands were detected by chemiluminescence using lumiQ ElectroChemiLuminescence (SB-WB012, ShareBio), and visualized and processed by the Molecular Imager ChemiDoc^TM^ XRS+ system with Image Lab^TM^ software (BioRad, USA). Between each step, membranes were washed three times with PBST.

### RNA isolation and quantitative real-time polymerase chain reaction

PDAC tissue samples were cut into small pieces and cryogenically ground before RNA extraction. Total RNA was extracted from cell lines and PDAC tissues using RNAiso PLUS (9109, Takara, USA). Unqualified RNA was excluded from further experiments (260/280 nm optical density ratio less than 1.85 or greater than 2.15, RNA concentration less than 100 ng/µL). Reverse transcription was conducted using HyperScript RT SuperMix (K1074, APExBIO, USA).

Quantitative real-time polymerase chain reaction (qRT–PCR) was performed using 2×Universal SYBR Green qPCR Premix (SB-Q204, ShareBio) on ViiA^TM^ 7 Real-Time PCR system (Applied Biosystems, USA). All reactions were run in triplicate. Primers were designed by Primer Premier, and their specificity was verified using Basic Local Alignment Search Tool (https://blast.ncbi.nlm.nih.gov/Blast.cgi). Melting curve analysis was conducted to further validate primer specificity. Primers generating single-peak melting curves were considered target-specific and thus used in subsequent assays. ACTB was used as an endogenous control, and the relative expression of target gene was determined by calculating 2^−ΔCt^, where the Ct of target gene was normalized to the CT of ACTB from the same individual sample. Primer sequences were listed in Table S1.

### Adiponectin secretion

Adiponectin concentrations in cell culture supernatants were quantified using ADP/Acrp30 ELISA Kit (E-EL-M0002, Elabscience, China) according to the manufacturer’s instructions and normalized to GAPDH expression (quantified by immunoblotting). Absorbances at 450 nm were measured using SYNERGY neo2 microplate reader (BioTek, USA).

### Lipolysis analysis

Cells were treated with isoproterenol at a final concentration of 100 nM for 30 min before supernatant harvest. Glycerol release was quantified using Lipolysis Colorimetric Assay Kit (E-BC-K798-M, Elabscience) according to the manufacturer’s instructions and normalized to GAPDH expression. Absorbances were measured at 550 nm.

### Cell proliferation assay

Cells were seeded in 96-well plates (1 × 10^4^ cells per well) with 30 replicates per group. Cell Count Kit-8 (SB-CCK8, ShareBio) was added to five replicate wells for continuous six days, followed by incubation at 37 °C for one hour. Absorbances at 450 nm were then measured using the microplate reader. Additionally, on day 6, three replicate wells were harvested, and cell numbers were counted respectively by Cellometer Mini (Nexcelom, USA).

### Cell cycle assay

Cells were seeded in 6-well plates (2 × 10^5^ cells per well) with three replicates per group. At each designated time point, cells from the three replicate wells were harvested and fixed by 75% ethanol overnight at −20 °C. Cells were then stained using Cell Cycle and Apoptosis Kit (SB-C6031, ShareBio) and subsequently analyzed by LSRFortessa^TM^ Flow Cytometry (BD Biosciences, USA).

### Transwell migration and invasion assays

For Transwell invasion assays, the Transwell upper chambers were pre-coated with Matrigel Basement Membrane Matrix, LDEV-free (354234, Corning). A total of 1 × 10^5^ cells in 200 µL FBS-free medium were seeded into each upper chamber of the 6.5 mm Transwell with 8.0 µm Pore Polycarbonate Membrane Insert (3433, Corning, USA). Then 600 µL complete medium was added to each lower chamber as a chemoattractant. After 24 h, the Transwell chambers were fixed with 4% paraformaldehyde, stained with Crystal Violet (C0121, Beyotime), and visualized using Axio Observer.

### Multi-omics sequencing and analysis

RNA quantity was analyzed using Qubit 4.0 (ThermoFisher), and quality was examined by electrophoresis on a denaturing agarose gel. ATAC sequencing and RNA sequencing were performed on the Illumina NovaSeq 6000 platform (Shanghai Xu Ran Biotechnology) using the 150 bp paired-end strategy. Raw reads were quality-trimmed by Skewer v0.2.2 [52] and accessed by FastQC v0.11.2 [53]. Clean reads were aligned to the human reference genome GRCh38, ensembled by STAR [54], and transcript quantification was conducted by StringTie v1.3.1c [55]

DAR and DEG were analyzed by DESeq2 v1.16.1 [56]. DARs and DEGs were subjected to enrichment analysis based on KEGG database [57] and GO terms using TopGO [58]. Significantly enriched terms were defined as those with *p* values less than 0.05. Aggregated footprinting analysis was performed using TOBIAS [59], with the detailed results provided in Tables S2 and S3.

All of the raw sequencing data were uploaded to the Sequence Read Archive (SRA), National Library of Medicine (NCBI, ncbi.nlm.nih.gov), and are accessible through Bioproject accession number PRJNA1393851.

### Statistical analysis

Integrated intensity and positive area of staining results were quantified by Image J. Statistical analysis was performed using SPSS, and graphs were generated using GraphPad Prism.

Data were presented as the means ± standard deviation. Shapiro–Wilk test was used to assess the normality for the data distribution, *F*-test was used to examine the homogeneity of variances, and the Student’s *t*-test was used for comparison between groups. Results were considered statistically significant at *p* ≤ 0.05, and marginally significant when 0.05 < *p* ≤ 0.01.

## Supplementary information

Reproducibility Checklist

Supplementary Figure 1

Supplementary Figure 2

Supplementary Figure 3

Supplementary Figure 4

Supplementary Figure 5

Supplementary Figure 6

Table S1. Primers used for qRT-PCR in this study.

Table S2. TOBIAS-results-significant

Table S3. TOBIAS-results-nonsignificant

Original Western blots

Supplementary figures legends

## Data Availability

The datasets used and analyzed during this study are available from the corresponding author on reasonable request.
