# Supplementary figures and images for: Adipogenic transdifferentiation reprograms EMT-high PDAC cells into a post-mitotic adipocyte-like state and limits metastasis
Source: Cell Death Dis. 2026 Mar 20;17(1):330. doi: 10.1038/s41419-026-08613-4 (PMC13039381; doi:10.1038/s41419-026-08613-4)

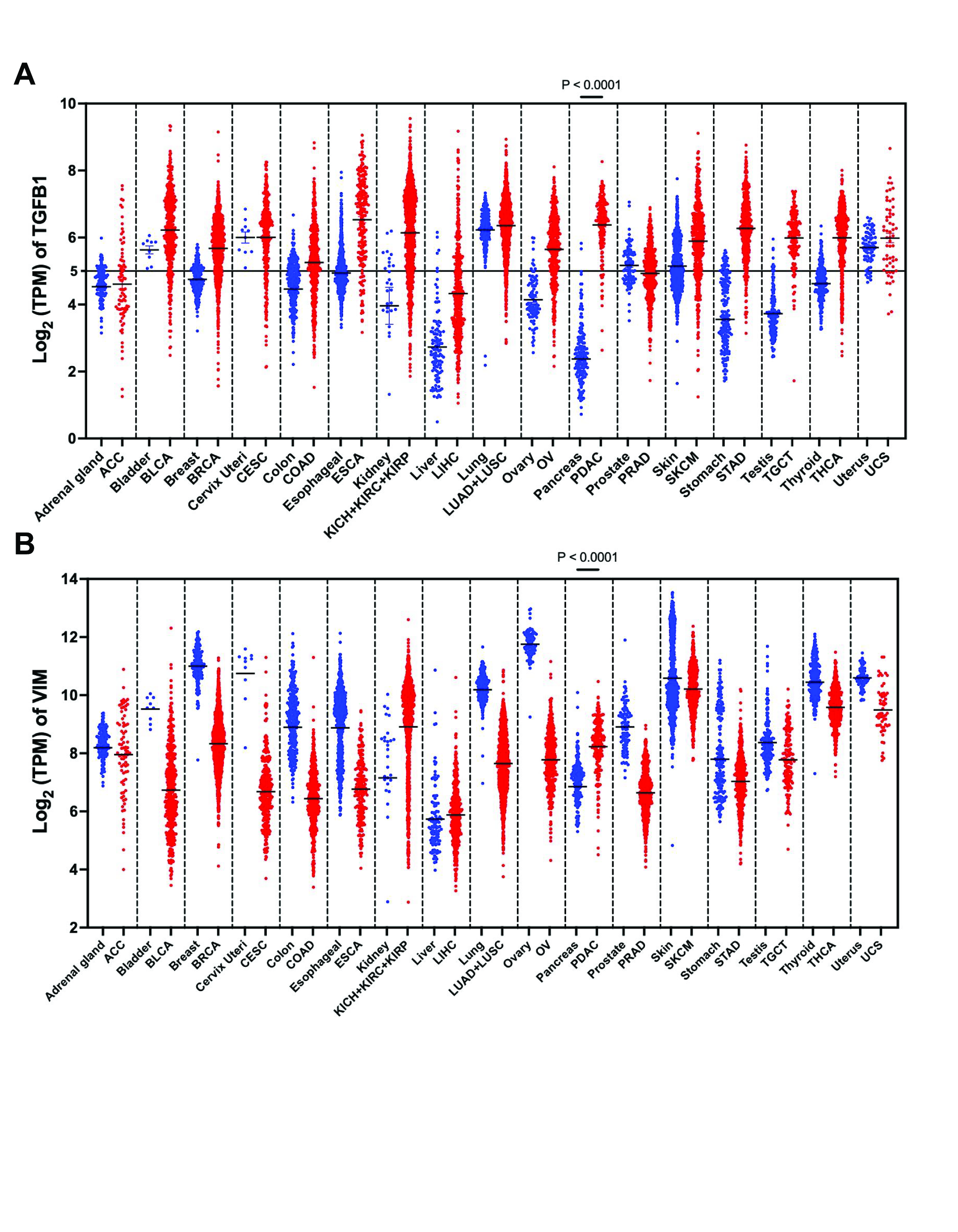

Supplement: Supplementary file 2 — Supplementary Figure 1 [file 41419_2026_8613_MOESM2_ESM.tif]

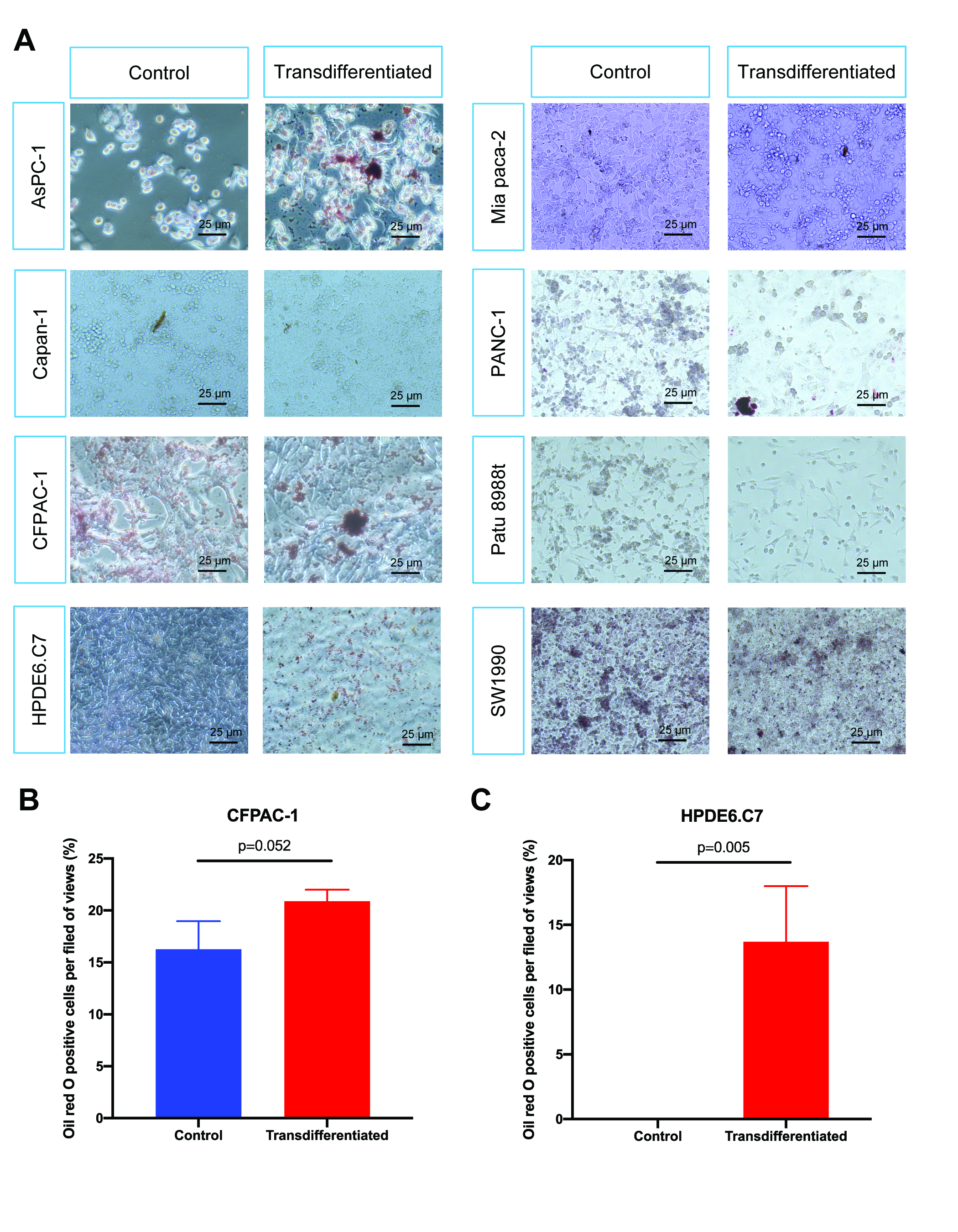

Supplement: Supplementary file 3 — Supplementary Figure 2 [file 41419_2026_8613_MOESM3_ESM.tif]

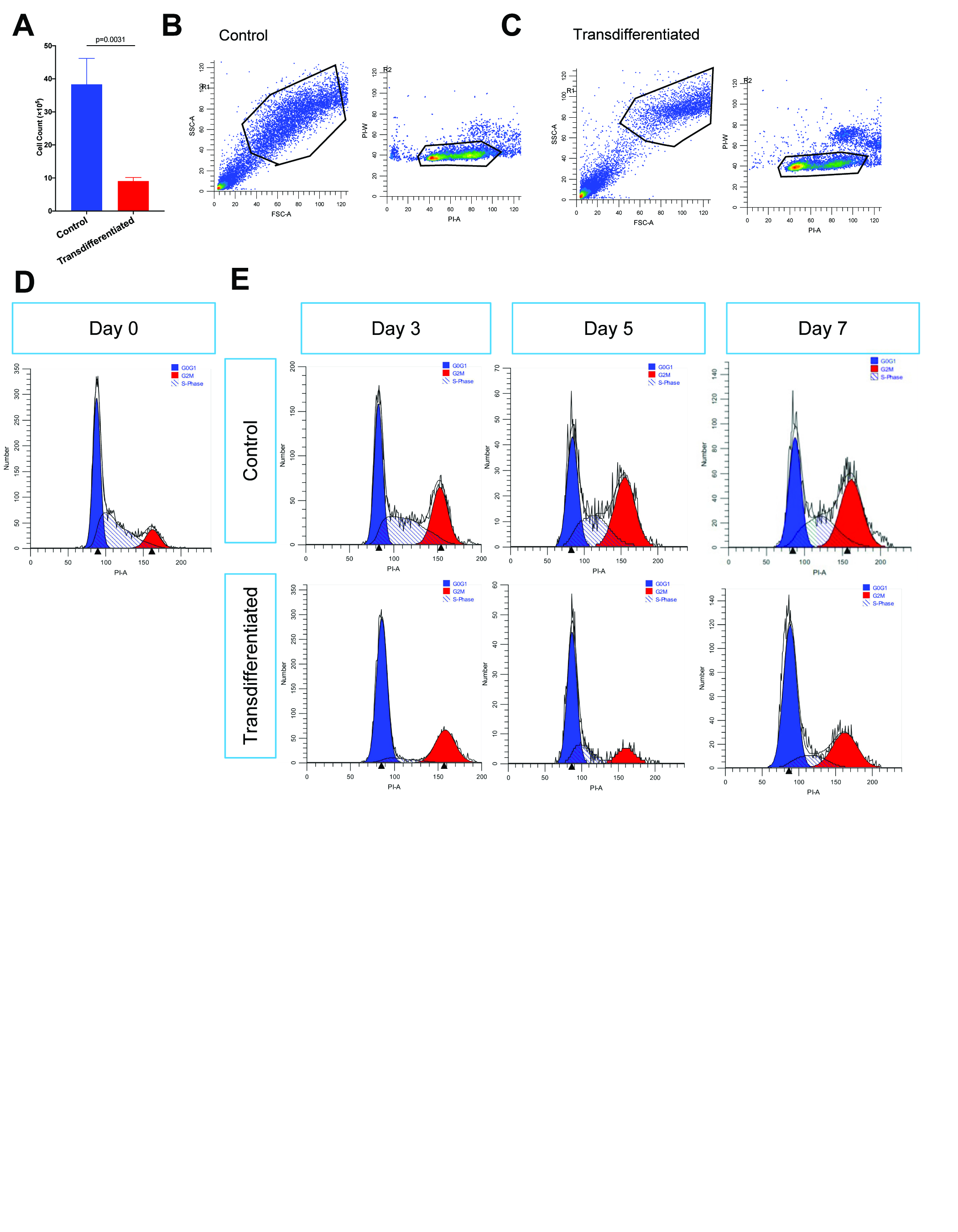

Supplement: Supplementary file 4 — Supplementary Figure 3 [file 41419_2026_8613_MOESM4_ESM.tif]

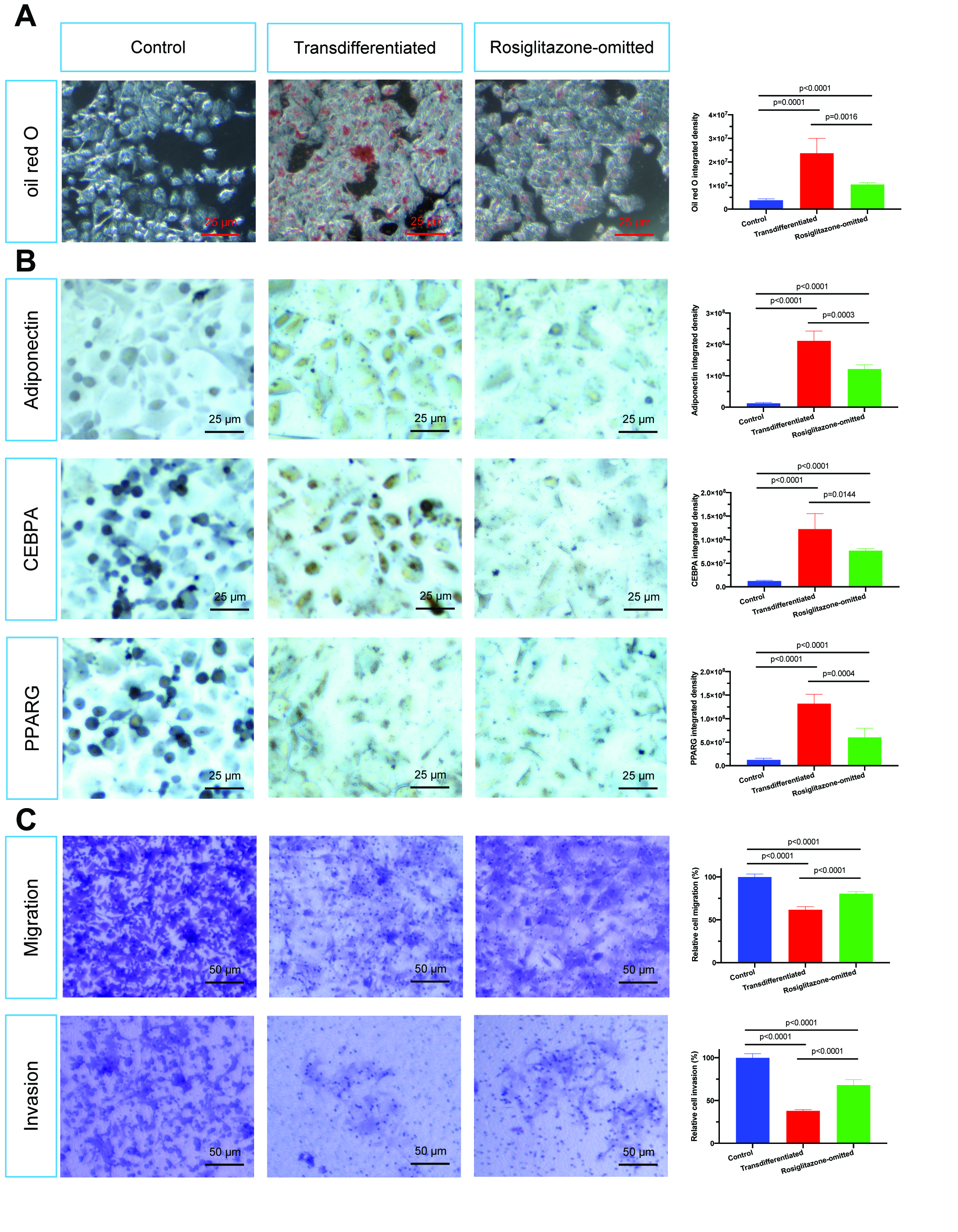

Supplement: Supplementary file 5 — Supplementary Figure 4 [file 41419_2026_8613_MOESM5_ESM.tif]

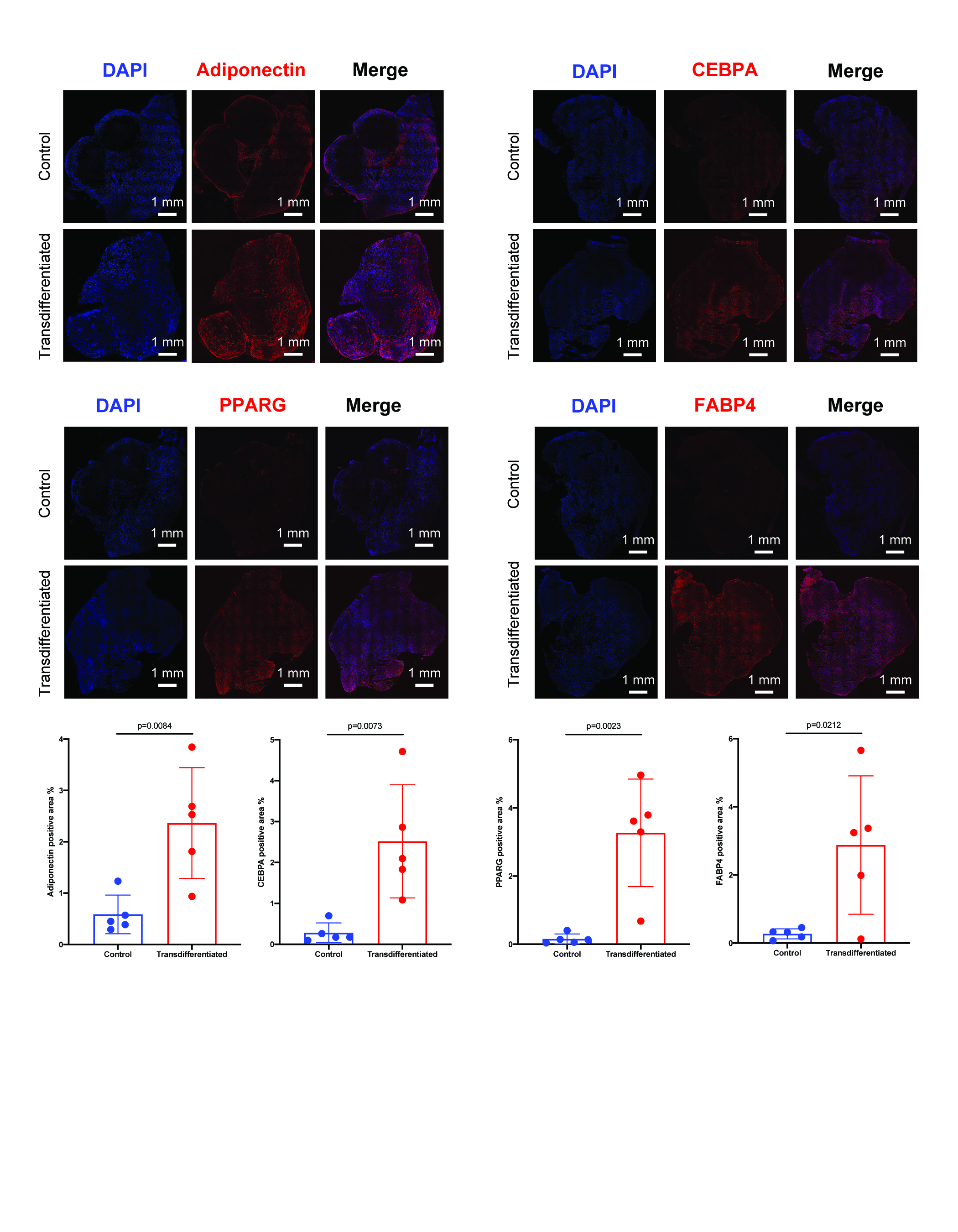

Supplement: Supplementary file 6 — Supplementary Figure 5 [file 41419_2026_8613_MOESM6_ESM.tif]

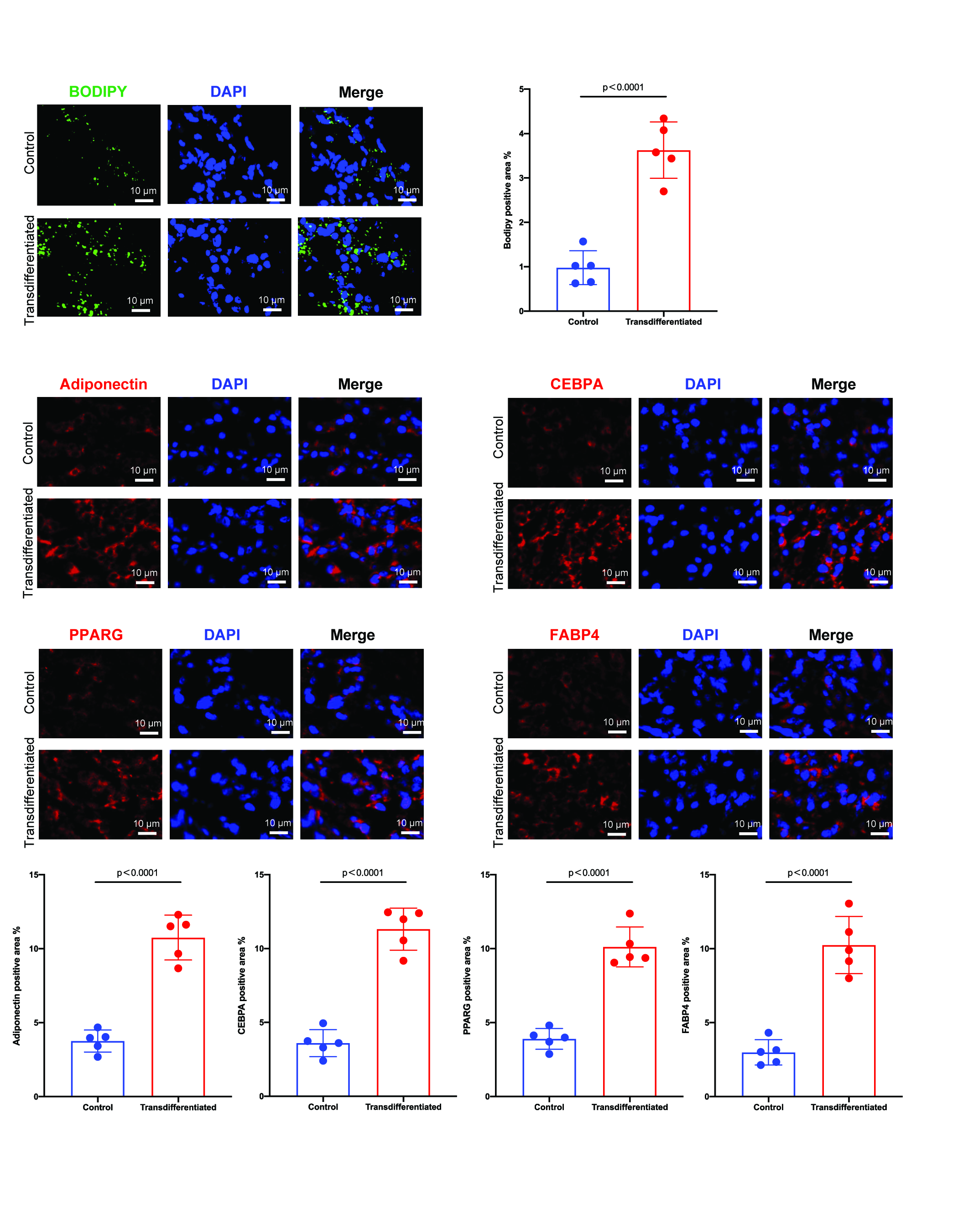

Supplement: Supplementary file 7 — Supplementary Figure 6 [file 41419_2026_8613_MOESM7_ESM.tif]

CEBPA

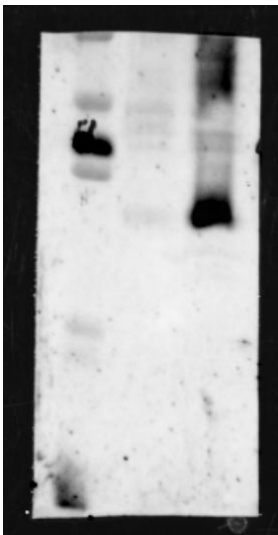

PPARG

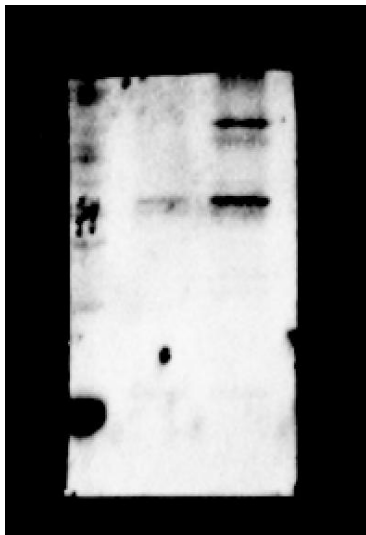

GAPDH

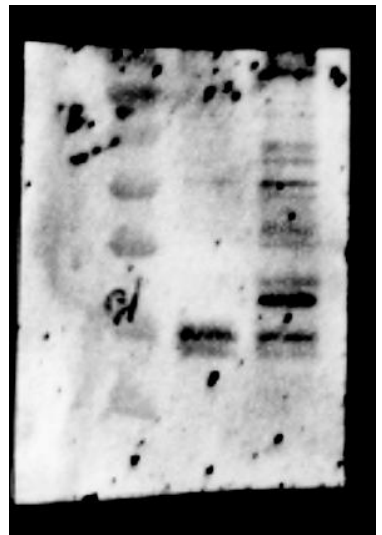

Supplement: Supplementary file 11 — Original Western blots [file 41419_2026_8613_MOESM11_ESM.pdf]
