# Supplementary material for: Adipogenic transdifferentiation reprograms EMT-high PDAC cells into a post-mitotic adipocyte-like state and limits metastasis
Source: Cell Death Dis. 2026 Mar 20;17(1):330. doi: 10.1038/s41419-026-08613-4 (PMC13039381; doi:10.1038/s41419-026-08613-4)
Supplement: Supplementary file 8 — Table S1. Primers used for qRT-PCR in this study. [file 41419_2026_8613_MOESM8_ESM.docx]

| Table S1. Primers used for qRT-PCR in this study. | | |
| --- | --- | --- |
| Gene name | Serial number | Primer sequence |
| ACTB | 1.F | 5-CGTGCGTGACATTAAGGAAGAGT-3 |
|  | 1.R | 5-GGAAGGAAGGCTGGAAGAGT-3 |
| Adiponectin | 1.F | 5-TTGCCTACCACATCACAGTCTA-3 |
|  | 1.R | 5-AGTCCATTACGCTCTCCTTCC-3 |
| CEBPA | 1.F | 5-CCTTCAACGACGAGTTCCTG-3 |
|  | 1.R | 5-GCTTGGCTTCATCCTCCTC-3 |
| PPARG | 1.F | 5-TCCACATTACGAAGACATTCCA-3 |
|  | 1.R | 5-CTCCACAGACACGACATTCAA-3 |
| FABP4 | 1.F | 5-AAGTCAAGAGCACCATAACCTT-3 |
|  | 1.R | 5-ACGCATTCCACCACCAGTT-3 |
| E-cadherin | 1.F | 5-ATGCCGCCATCGCTTACA-3 |
|  | 1.R | 5-GCTGTTGCTGTTGTGCTTAAC-3 |
| Vimentin | 1.F | 5-CAGGAGGAGATGCTTCAGAGA-3 |
|  | 1.R | 5-CGTGAGGTCAGGCTTGGAA-3 |
| Snail1 | 1.F | 5-CGCTGCCAATGCTCATCTG-3 |
|  | 1.R | 5-AGTAGAGGAGAAGGACGAAGGA-3 |
| Snail2 | 1.F | 5-TGCCTGTCATACCACAACCA-3 |
|  | 1.R | 5-TGGAGGAGGTGTCAGATGGA-3 |
| Twist1 | 1.F | 5-TGAGCAACAGCGAGGAAGA-3 |
|  | 1.R | 5-TCGTAAGACTGCGGACTCC-3 |
| Twist2 | 1.F | 5-CAAGCTCAGCAAGATCCAGAC-3 |
|  | 1.R | 5-TGTCCATCTCGTCGCTCTG-3 |
| ZEB1 | 1.F | 5-AGTGGCGGTAGATGGTAATGTA-3 |
|  | 1.R | 5-AGGCTGCTCAAGACTGTAGTT-3 |
| ZEB2 | 1.F | 5-GCTGAGGATGACGGTATTGC-3 |
|  | 1.R | 5-GGCTTGTAGAATCTCGTTGTTG-3 |
| F: forward, R: reverse |  |  |
