# Supplementary material for: Adipogenic transdifferentiation reprograms EMT-high PDAC cells into a post-mitotic adipocyte-like state and limits metastasis
Source: Cell Death Dis. 2026 Mar 20;17(1):330. doi: 10.1038/s41419-026-08613-4 (PMC13039381; doi:10.1038/s41419-026-08613-4)
Supplement: Supplementary file 12 — Supplementary figures legends [file 41419_2026_8613_MOESM12_ESM.docx]

Figure S1. PDAC displays significantly increased expression of EMT-relevant markers.

Comparison analysis of gene expression of EMT inducer TGF-b (A) and mesenchymal marker vimentin (B) in normal epithelial organs and corresponding tumors based on TCGA and GTEx databases. Statistical significance was evaluated by student’s t test.

Abbreviation: ACC: Adrenocortical carcinoma, BLCA: Bladder Urothelial Carcinoma, BRCA: Breast invasive carcinoma, CESC: Cervical squamous cell carcinoma and endocervical adenocarcinoma, COAD: Colon adenocarcinoma, ESCA: Esophageal carcinoma, KICH: Kidney Chromophobe, KIRC: Kidney renal clear cell carcinoma, KIRP: Kidney renal papillary cell carcinoma, LIHC: liver hepatocellular carcinoma, LUAD: Lung adenocarcinoma, LUSC: Lung squamous cell carcinoma, OV: Ovarian serous cystadenocarcinoma, PDAC: pancreatic ductal adenocarcinoma, PRAD: Prostate adenocarcinoma, SKCM: Skin cutaneous melanoma, STAD: Stomach adenocarcinoma, TGCT: Testicular germ cell tumors, THCA: Thyroid carcinoma, UCS: Uterine carcinosarcoma, EMT: Epithelial-mesenchymal transition.

Figure S2. AsPC-1, CFPAC-1 and HPDE6.C7 displayed obvious adipogenesis after transdifferentiation induction.

A: Visualization of accumulated lipid droplets in control and transdifferentiated PDAC cell lines (AsPC-1, Capan-1, CFPAC-1, Mia PaCa-2, PANC-1, PaTu 8988t, SW-1990) and a normal pancreatic cell line (HPDE6.C7) on day 10 by oil red O staining.

B, C: Quantification of the percentage of oil red O positive cells in five random field of views of CFPAC-1 (B) and HPDE6.C7 cells (C). Values were presented as the mean ± SD (n=5 per group) and compared by student’s t test.

Figure S3. Adipogenic transdifferentiation of AsPC-1 arrested cell cycle at post-miotic phase.

A: Cell numbers in control and transdifferentiated groups on day 6 after induction (9.09±1.05 ×10^5^ versus 38.35±7.84 ×10^5^ in control, p=0.0031). Values were presented as the mean ± SD (n=3 per group) and compared by student’s t test.

B, C: FSC signals in flow cytometry gates of control (B) and transdifferentiated (C) groups. The singlet cells were separated in the PI-W and PI-A window. The rightward shift in FSC distribution of the transdifferentiated group indicates an increase in cell size.

D: Cell cycle analysis of AsPC-1 on day 0 by flow cytometry.

E: Cell cycle analysis of control and transdifferentiated AsPC-1 on day 3, 5 and 7 after induction by flow cytometry.

Abbreviation: SD: Standard deviation; FSC: Forward scatter; PI-W: Propidium iodide width; PI-A: Propidium iodide area.

Figure S4. Rosiglitazone plays a partial but not exclusive role in adipogenic transdifferentiation.

Comparison of control group, transdifferentiated group and rosiglitazone-omitted group was performed using oil red O staining (A), immunostaining for the adipocyte specific marker adiponectin and the transcription factors CEBPA and PPARG (B), and Transwell assays (C). Five random field of views were used for quantification, values were presented as the mean ± SD (n=5 per group) and compared by student’s t test.

Abbreviation: SD: Standard deviation.

Figure S5. Adipogenic transdifferentiation treatment-induced orthotopic PDAC tumors expressed adipocyte markers.

Immunofluorescence visualization and quantification of adiponectin, CEBPA, PPARG, and FABP4 in control (up) and transdifferentiated groups (down) from orthotopic PDAC samples. DAPI was used as nucleus indicator. Five random field of views were used for quantification, values were presented as the mean ± SD (n=5 per group) and compared by student’s t test. Corresponding high power fields were displayed in Figure S6.

Figure S6. Representative magnified views of fluorescence staining of lipid droplets, adiponectin, CEBPA, PPARG and FABP4.

Five high power fields were used for quantification, values were presented as the mean ± SD (n=5 per group) and compared by student’s t test.

Abbreviation: SD: Standard deviation.
